# Antibacterial Activity of *Cinnamomum camphora* Essential Oil on *Escherichia coli* During Planktonic Growth and Biofilm Formation

**DOI:** 10.3389/fmicb.2020.561002

**Published:** 2020-11-12

**Authors:** Lei Wang, Kang Zhang, Kai Zhang, Jingyan Zhang, Jingjing Fu, Jie Li, Guibo Wang, Zhengying Qiu, Xuezhi Wang, Jianxi Li

**Affiliations:** Key Lab of Veterinary Pharmaceutical Development, Ministry of Agriculture and Rural Affairs, Engineering and Technology Research Center of Traditional Chinese Veterinary Medicine, Gansu Province, Lanzhou Institute of Husbandry and Pharmaceutical Sciences of Chinese Academy of Agricultural Sciences, Lanzhou, China

**Keywords:** *Cinnamomum camphora* essential oil, bactericidal effect, *Escherichia coli*, planktonic growth, biofilm

## Abstract

Bacterial biofilms are believed to be principal virulence factors for many localized chronic infectious diseases. *Escherichia coli* is one of the most common microbial pathogens and frequently causes biofilm-associated opportunistic infections, such as diarrhea, endometritis and mastitis. *Cinnamomum camphora* essential oil (CCEO) has shown potential in treating intractable chronic endometritis in dairy cows. There is little scientific evidence regarding the effect of CCEO on bacterial biofilms. The objective of this study was to investigate the effect of CCEO on *E. coli* biofilm formation and how CCEO affects *E. coli* in suspension and in a biofilm. CCEO killed all clinical *E. coli* strains in either planktonic or biofilm state isolated from dairy cows with clinical endometritis. The minimum inhibitory concentration (MIC) for 90% of the organisms was 4.297 μL/mL, the minimum bactericidal concentration for 90% of the organisms was 6.378 μL/mL, the minimum biofilm inhibitory concentration for 90% of the organisms was 6.850 μL/mL, and the minimum biofilm eradication concentration (MBEC) for 90% of the organisms was 8.467 μL/mL. The MBECs were generally two times higher than the MICs. Flow cytometry analysis confirmed that significant bacterial killing occurred during the first 1 h after exposure to subinhibitory concentrations of CCEO. In addition, CCEO exerted a significant inhibitory effect on *E. coli* biofilm formation, and bacterial killing occurred during the first 30 min of exposure to subinhibitory biofilm concentrations of CCEO. The biofilm yield of *E. coli* was significantly reduced after CCEO treatment, along with an increased dead/live microbial ratio in biofilms compared with that in the non-treated control, as confirmed by scanning electron microscopy images and confocal laser scanning microscopy images. These data revealed that CCEO efficiently kills *E. coli* during planktonic growth and biofilm formation.

## Introduction

Bacterial biofilms, an emergent form of bacterial life, are “aggregates of microorganisms in which cells are frequently embedded in a self-produced matrix of extracellular polymeric substances that are adherent to each other and/or a surface” (Vert et al., 2012; Flemming et al., 2016). The National Institutes of Health revealed that 65% of all microbial infections and 80% of all chronic infections are associated with biofilm formation. Microorganisms in biofilms develop elevated resistance to antimicrobial agents and host defense systems through the physical barrier of the extracellular matrix, metabolic dormancy or molecular persistence programs (Van Acker et al., 2014). During the dispersion of a biofilm, the microbial cells within the biofilm quickly proliferate and disperse to switch from a sessile to a motile form. Detachment then occurs in a natural pattern (Costerton et al., 1999). The formation of bacterial biofilms increases the hardiness of the bacteria and contributes to their persistence during infection, posing great challenges in the use of conventional antimicrobials. Nonetheless, the control of biofilm formation and treatment of existing biofilms remains tenuous, with few new therapeutic options currently available for clinical use. Targeting microbial biofilms is a current and prospective therapeutic strategy (Koo et al., 2017).

The essential oil of *Cinnamomum camphora* (L.) Presl (CCEO) has a broad range of antimicrobial, insecticidal, anti-inflammatory and antioxidant activities. CCEO contains 330 different compounds, including linalool and camphor, which are the main antibacterial components (Zhang et al., 2019). Many studies have been carried out on the antibacterial activity of CCEO, revealing, for example, activities against *Escherichia coli*, *Staphylococcus aureus*, and *Choanephora cucurbitarum* (Pragadheesh et al., 2013; Zhang et al., 2014; Wu et al., 2019). Preliminary clinical trials have shown that the cure rate of dairy cows suffering from resistant endometritis or chronic endometritis treated with chloramphenicol and furacilin oil increases significantly when 20% camphor oil has been added (Meng et al., 1984). Our previous studies in the lab of traditional Chinese veterinary medicine in Lanzhou Institute of Husbandry and Pharmaceutical Sciences of Chinese Academy of Agricultural Sciences also showed that 35 of 48 cows recovered from endometritis following treatment with 4% CCEO (sweet almond oil as solvent) under field conditions (data not published). However, there is little scientific evidence indicating that this bioactivity is associated with the inhibition of bacterial biofilm formation by CCEO. *E. coli* is one of the most common microbial pathogens and frequently causes biofilm-associated opportunistic infections, such as endometritis (Ferris et al., 2016). We speculated that CCEO inhibits *E. coli* in biofilms, which contributes to the cure of refractory endometritis. Therefore, we studied the effect of CCEO on planktonic growth and biofilm formation by *E. coli* isolated from dairy cows suffering from clinical endometritis and how CCEO affects *E. coli* in suspension and in a biofilm.

## Materials and Methods

### Materials

*Cinnamomum camphora* essential oil was purchased from Jiangxi Huitong Pharmaceutical Fragrance Oil Co., Ltd., and analyzed via gas chromatography-mass spectrometry. Thirty-one compounds were identified, constituting 70.04% (v/v) of the oil. The major compound was linalool (17.98%, v/v), followed by camphor (17.15%, v/v), eucalyptol (12.71%, v/v), and alpha-terpineol (3.43%, v/v).

### Bacterial Strains

Forty-four clinical strains of *E. coli* were isolated from the intrauterine mucus of Holstein cows affected with clinical endometritis 21–50 days after calving, and the cows were collected from five farms located in Gansu Province, Shaanxi Province, and Qinghai Province in Northwest China. Then, these clinical strains were identified as *E. coli* using colony morphology analysis on blood agar, Gram staining, biochemical identification and 16S rDNA sequencing. Accession numbers of their 16S rRNA sequences in GenBank are KJ57728-KJ5772872 and MW025989-MW026027. *E. coli* ATCC 25922 was purchased from the American Type Culture Collection. Biofilm formation of *E. coli* was determined by the crystal violet method. All the strains were stored at −80°C in a microbiology laboratory located in the Lanzhou Institute of Husbandry and Pharmaceutical Sciences of Chinese Academy of Agricultural Sciences, Lanzhou, China.

### Determination of the Minimum Inhibitory Concentration (MIC) and Minimum Bactericidal Concentration (MBC)

The minimum inhibitory concentration (MIC) and minimum bactericidal concentration (MBC) of CCEO against forty-four *E. coli* isolates and *E. coli* ATCC25922 were determined using the broth microdilution method according to Kwieciński et al. (2009) with minor modifications. *E. coli* was incubated (37°C, shaking) in Mueller-Hinton (MH) broth until the exponential growth phase was reached. A diluted bacterial suspension was added to a 96-well microtiter plate at a final concentration of 1 × 10^5^ CFU/mL based on a turbidity comparator (DensiCHEK plus, BioMerieux SA, France). Serial twofold dilutions of CCEO were prepared and added to each well to obtain a final concentration range from 0.25 to 128 μL/mL. All wells contained 1% DMSO (v/v) to enhance the solubility of CCEO. In addition, there were solvent control (test bacteria and MH broth containing 1% DMSO), bacterial control (test bacteria and MH broth), blank control (MH broth containing 1% DMSO and corresponding concentrations of CCEO), blank solvent control (MH broth containing 1% DMSO) and blank medium (MH broth). All plates were incubated at 37°C for 24 h, and growth was evaluated by the turbidimetric method. The MIC was defined as the lowest CCEO concentration at which no visible growth was detected (optical density at 600 nm (OD_600_ nm) ≤ 0.05).

From the wells representing the MIC and the three next highest concentrations, 10 μL of the test solutions was removed and plated on MH agar, and the plates were incubated at 37°C for 24 h. Finally, the number of colonies on the agar was counted. The MBC was defined as the lowest concentration at which no colonies were observed. The MIC and MBC were determined for all 44 clinical *E. coli* strains and *E. coli* ATCC 25922. For each strain, at least three replicates were analyzed, and the modal value was determined.

### Determination of the Minimum Biofilm Inhibitory Concentration (MBIC) and Minimum Biofilm Eradication Concentration (MBEC)

The minimum biofilm inhibitory concentration (MBIC) of CCEO against 39 *E. coli* isolates and *E. coli* ATCC25922 was determined using the microdilution method with minor modifications (AI-Shabib et al., 2017). A bacterial suspension (2 × 10^7^ CFU/mL) in Luria-Bertani (LB) broth was added to a 96-well microtiter plate (100 μL per well) with serial twofold dilutions of CCEO (0.5 to 256 μL/mL, 100 μL per well). All wells contained a final DMSO concentration of 1% (v/v). In addition, there were solvent control (test bacteria and LB broth containing 1% DMSO), bacterial control (test bacteria and LB broth), blank control (LB broth containing 1% DMSO and corresponding concentrations of CCEO), blank solvent control (LB broth containing 1% DMSO) and blank medium (LB broth). All plates were incubated at 37°C for 24 h, and then, the medium was aspirated. Each well was washed three times with PBS, dried, fixed with 200 μL of methanol for 15 min, stained with 0.3% (w/v) crystal violet for 5 min, and rinsed with deionized water. Subsequently, 200 μL of 33% (v/v) glacial acetic acid was added to the wells. Finally, the plates were shaken at room temperature for 5 min, and the OD_600 *nm*_ was measured using a microplate reader (SpectraMax M2^*e*^, Molecular Devices, United States). The MBIC was defined as the lowest concentration of CCEO that resulted in at least 90% inhibition of biofilm formation compared with that in the control without CCEO.

The minimum biofilm eradication concentration (MBEC) of CCEO against *E. coli* was determined by the microdilution method with minor modifications (Ramage et al., 2001). Two hundred microliters of bacterial suspension (1 × 10^7^ CFU/mL) in LB broth was added to each well in a 96-well microtiter plate for biofilm formation. After incubation at 37°C for 24 h, the medium was aspirated, and each well was washed three times with PBS. Subsequently, serial twofold dilutions of CCEO (0.25 to 128 μL/mL) were added to the wells containing biofilms. All wells contained a final DMSO concentration of 1% (v/v). In addition, solvent control (biofilm and LB broth containing 1% DMSO), biofilm control (biofilm and LB broth), blank control (LB broth containing 1% DMSO and corresponding concentrations of CCEO), blank solvent control (LB broth containing 1% DMSO) and blank medium (LB broth) were also performed. The plates were incubated at 37°C for 24 h, the medium was aspirated, and each well was washed three times with PBS. Subsequently, the biofilms were stained with 0.3% (w/v) crystal violet and rinsed with deionized water. Finally, 33% (v/v) glacial acetic acid was added, and measurements were made with the method described above. The MBEC was defined as the lowest CCEO concentration that resulted in at least 80% eradication of biofilms compared with that in the control without CCEO.

### Planktonic Time-Dependent Killing Assay

In test tubes, *E. coli* ATCC 25922 suspensions (1 × 10^5^ CFU/mL) were mixed with CCEO at a final concentration of 0 (control), 1, 2, 4, or 8 μL/mL and with 1% DMSO (v/v) in all tubes. All tubes were incubated at 37°C with shaking. After 5, 15, and 30 min and 1, 2, 4, 8, 12, and 24 h, 100 μL was removed from each tube, serially diluted, plated on MH agar and incubated at 37°C for 24 h. The number of viable *E. coli* cells was determined by counting the colonies formed. The detection limit was 10 CFU/mL. A sample from the 0 μL/mL CCEO tube taken immediately after mixing was used as a “time 0” control. Measurements were performed in three independent experiments. Time-kill curves were constructed by plotting the mean colony counts (Log_10_ CFU/mL) versus time.

### Flow Cytometry (FCM) Assay

The effect of CCEO on *E. coli* ATCC 25922 cell viability during planktonic growth was analyzed by flow cytometry (FCM) with minor modifications (Kang et al., 2018). *E. coli* ATCC 25922 suspensions (1 × 10^6^ CFU/mL) were treated with CCEO at 0 μL/mL (control), 2, 4, and 8 μL/mL for 0.5, 1, 4, and 24 h at 37°C. The samples were centrifuged at 10000 rpm for 5 min, the medium was removed, and the cells were resuspended in physiological saline. Then, the cells were stained with SYTO 9 and propidium iodide (PI) dyes from the LIVE/DEAD BacLight Bacterial Viability Kit (Molecular Probes, Invitrogen, France) in the dark for 15 min. The samples were centrifuged again at 10000 rpm for 5 min, the supernatant fluid was removed, and the cells were resuspended in physiological saline. FCM (Cytomics FC 500, Beckman Coulter, United States) was used to determine the viability of the *E. coli* cells. The acquisition time/events of the protocol were 300 s/100000 cells.

### Biofilm Time-Dependent Killing Assay

A bacterial suspension of *E. coli* ATCC 25922 (2 × 10^7^ CFU/mL) in LB broth was added to a 96-well microtiter plate (100 μL per well). CCEO solutions (0, 2, 4, 8, and 16 μL/mL) in 100 μL of LB with 1% DMSO (v/v) were also added to the wells. The plates were incubated at 37°C for 5, 15, 30, 60, 120, 240, and 480 min. The medium was then aspirated, the wells were washed three times with PBS, and 200 μL of LB was added to each well. Then, 40 μL of CCK-8 solution from the Cell Counting Kit-8 assay (CCK-8, Beyotime Biotechnology, China) was added to the wells with the biofilms. Following incubation at 37°C for 2 h, the absorbance at 450 nm was recorded. All measurements were performed in three independent experiments.

### Scanning Electron Microscopy (SEM) Analysis

The effects of CCEO on bacterial attachment and biofilm formation were examined using scanning electron microscopy (SEM) according to a previously described method with minor modifications (Kang et al., 2018). *E. coli* ATCC 25922 suspensions (1 × 10^7^ CFU/mL) were prepared in LB broth with CCEO concentrations of 0, 2, and 4 μL/mL. The samples were incubated to form biofilms on a glass coverslip (8 mm) in a 24-well polystyrene plate for 24 h without shaking at 37°C. The samples were fixed for 4 h using 2.5% glutaraldehyde, washed with PBS and dehydrated using a graded ethanol series (30, 50, 70, 85, 90, 100, and 100%; 15 min each). After drying, the samples were sputter-coated with gold and then imaged with SEM (JSM-5600, JEOL, Japan).

### Confocal Laser Scanning Microscopy (CLSM) Analysis

The effects of CCEO on viable bacteria during biofilm formation were examined using confocal laser scanning microscopy (CLSM). After the *E. coli* ATCC 25922 suspensions (1 × 10^7^ CFU/mL) were incubated with CCEO concentrations of 0 and 2 μL/mL for 24 h, the biofilms were stained with SYTO 9 and PI from the LIVE/DEAD BacLight Bacterial Viability Kit (Molecular Probes, Invitrogen, France). After staining in the dark for 15 min, the samples were washed with PBS and imaged with a CLSM (LSM 700, Zeiss, Germany) equipped with a 40× objective lens. The excitation/emission maxima were approximately 483/500 nm for the SYTO 9 stain and 305/617 nm for PI. Each sample took six fields, and three independent experiments were performed.

The images obtained from CLSM were quantified for biomass of dead cells and live cells with COMSTAT (Heydorn et al., 2000; Vorregaard, 2008)^1^.

### Statistical Analysis

Statistical analysis was performed with SPSS software, version 16.0 (SPSS, Inc., United States). One-way analysis of variance was performed to detect the significant effects of variables, followed by a Student–Newman–Keuls test. Differences were considered significant at *p* < 0.05.

## Results

### CCEO Has a Strong Bactericidal Effect and Disrupts Biofilms

**FIGURE 1:**
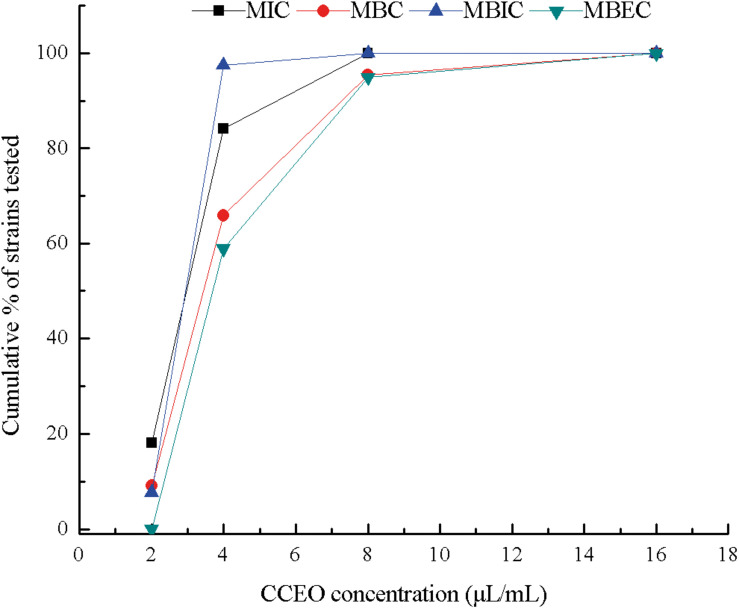
Cumulative minimum inhibitory concentration (MIC), minimum bactericidal concentration (MBC), minimum biofilm inhibitory concentration (MBIC), and minimum biofilm eradication concentration (MBEC) of *C. camphora* essential oil (CCEO) against clinical strains of *E. coli* isolated from dairy cows affected with clinical endometritis in China, expressed as a percentage of the tested strains. MIC and MBC, *n* = 44; MBIC and MBEC, *n* = 39.

From 44 *E. coli* isolates from dairy cows with endometritis in China, 39 *E. coli* isolates could form biofilms. CCEO exhibited significant bactericidal activity against *E. coli* and strong activity against the *E. coli* biofilm. The cumulative MIC, MBC, MBIC, and MBEC values of CCEO against the clinical isolates of *E. coli* are summarized in Figure 1. For the clinical isolates, the MICs ranged from 2 to 8 μL/mL, the MBCs ranged from 2 to 16 μL/mL, the MBICs ranged from 2 to 8 μL/mL, and the MBECs ranged from 4 to 16 μL/mL (details in Supplementary Table S1). The MIC for 50% of the organisms (MIC_50_) was 2.953 μL/mL, the MIC for 90% of the organisms (MIC_90_) was 4.297 μL/mL, and the MBC_50_ and MBC_90_ were 3.870 and 6.378 μL/mL, respectively. The MBIC_50_, MBIC_90_, MBEC_50_, and MBEC_90_ were 3.619, 6.850, 4.693, and 8.467 μL/mL, respectively. For *E. coli* ATCC 25922, the MIC, MBC, MBIC, and MBEC were 4, 8, 4, and 8 μL/mL, respectively.

### Effect of CCEO on Planktonic Growth and Cell Viability of *E. coli*

**FIGURE 2:**
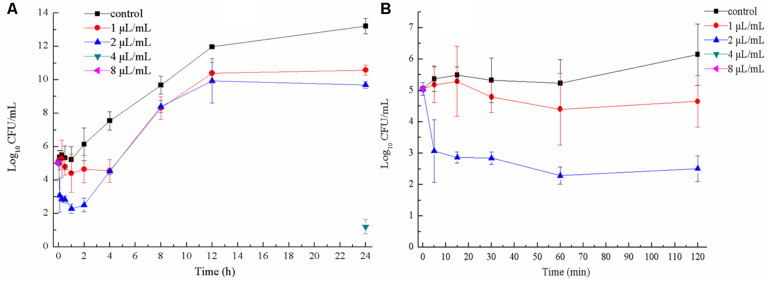
Time-kill curves of *C. camphora* essential oil (CCEO) at 0, 1, 2, 4, or 8 μL/mL against planktonic *E. coli* ATCC 25922 for 24 h **(A)** and for 2 h **(B)**. A sample of 0 μL/mL CCEO was used as a control. Data indicates confidence interval at 95% of three independent experiments.

When *E. coli* ATCC 25922 was exposed to 1 and 2 μL/mL CCEO for 1 h, the number of viable cells was the lowest (Figure 2A). After exposure to 1 μL/mL CCEO for 4 h, the inhibition of *E. coli* by CCEO was the strongest, and only 0.1% of the viable *E. coli* cells in the control without CCEO were observed in the exposed group. After treatment with 2 μL/mL CCEO, the number of viable *E. coli* cells was reduced by two log_10_ steps (99% killed) after 5 min (Figure 2B), and a reduction of three log_10_ steps (99.9% killed) was observed after 1 to 4 h (Figure 2A). Treatment with 4 μL/mL CCEO resulted in a reduction of five log_10_ steps (99.999% killed) after 5 min, and viable *E. coli* cells were detected (approximately 16.7 CFU/mL) until the addition of 4 μL/mL CCEO for 24 h. After treatment with 8 μL/mL CCEO, no viable *E. coli* was detected during the observation period. The rate of killing was dosage-dependent over the range of CCEO concentrations tested. The bactericidal activity of CCEO was related to the *E. coli* growth phase, with the lowest level in viable counts observed at approximately 1 h.

**FIGURE 3:**
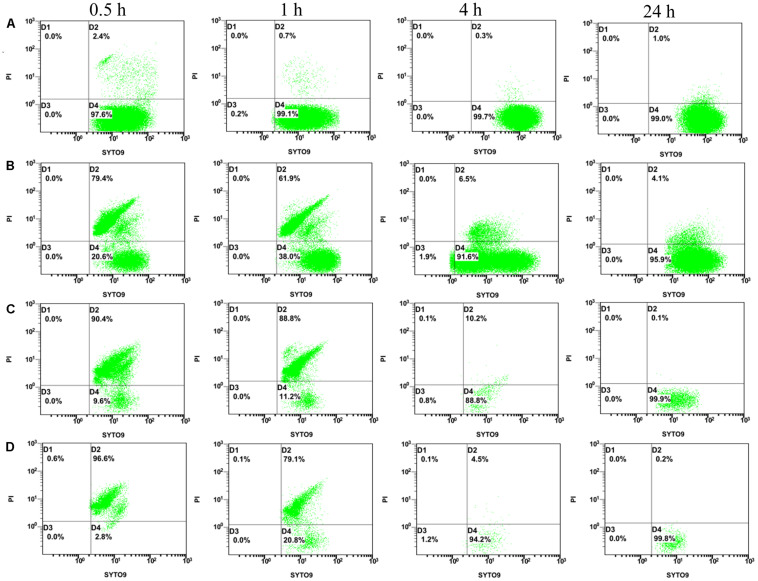
Flow cytometry (FCM) dot plots for *E. coli* ATCC 25922: **(A)** control; **(B)** treated with *C. camphora* essential oil (CCEO) at 2 μL/mL for 0.5, 1, 4, and 24 h; **(C)** treated with CCEO at 4 μL/mL for 0.5, 1, 4, and 24 h; and **(D)** treated with CCEO at 8 μL/mL for 0.5, 1, 4, and 24 h. A sample of 0 μL/mL CCEO was used as a control.

Flow cytometry dual-parameter dot plots of *E. coli* ATCC25922 treated with CCEO at 0, 2, 4, and 8 μL/mL and stained with SYTO 9 and PI are shown in Figure 3. More than 97% of the *E. coli* cells in the control group with 0 μL/mL CCEO were located in the D4 quadrant (Figure 3A), indicating that the untreated *E. coli* cells had intact cell membranes and were almost all viable. After treatment with 2 μL/mL CCEO, the number of cells was lowest at 1 h; moreover, only 98633 cells were acquired in 300 s at 1 h, 100000 cells were acquired in 267.8 s at 0.5 h, 100000 cells were acquired in 26.9 s at 4 h and 100000 cells were acquired in 23.9 s at 24 h (Figure 3B). The cells shown in Figures 3C,D were all acquired in 300 s, and the lowest values were observed at 4 h after treatment with 4 or 8 μL/mL CCEO. After exposure to CCEO, the number of cells dramatically decreased, and the number of viable cells decreased with increasing CCEO concentration. The proportion of viable cells increased over time.

### Effect of CCEO on *E. coli* Biofilm Formation

**FIGURE 4:**
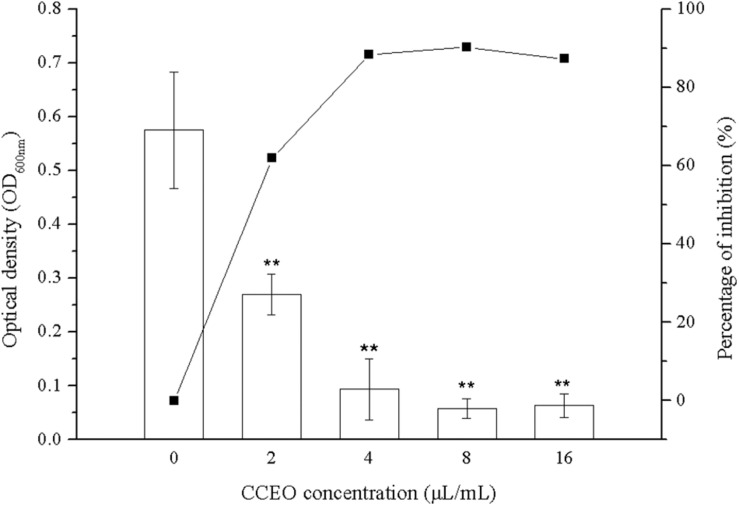
Effect of *C. camphora* essential oil (CCEO) on *E. coli* ATCC 25922 biofilms determined by crystal violet assays. A sample of 0 μL/mL CCEO was used as a control. Data represents the mean ± SD of three independent experiments, ** indicates *p* < 0.01 compared with the control group.

**FIGURE 5:**
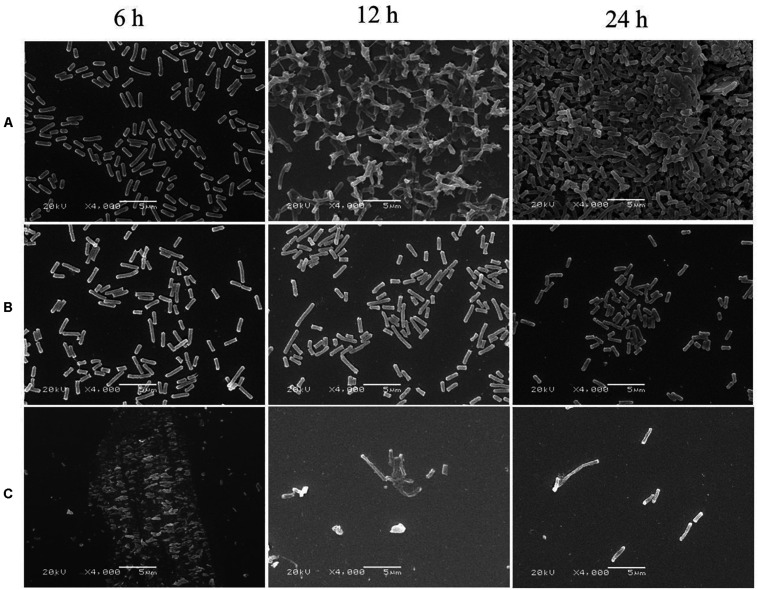
Scanning electron microscopy (SEM) images of *E. coli* ATCC 25922 biofilms: **(A)** control; **(B)** treated with *C. camphora* essential oil (CCEO) at 2 μL/mL for 6, 12, and 24 h; **(C)** treated with CCEO at 4 μL/mL for 6, 12, and 24 h. A sample of 0 μL/mL CCEO was used as a control.

Biofilm formation of *E. coli* ATCC 25922 was significantly inhibited after exposure to CCEO, as shown by quantitative crystal violet assays (Figure 4) and SEM analysis (Figure 5). CCEO at 2 μL/mL significantly inhibited biofilm formation (*p* < 0.01). Eighty-eight percent of *E. coli* biofilm formation was inhibited after treatment with 4 μL/mL CCEO, and an increase in the CCEO concentration did not further inhibit biofilm formation (Figure 4). SEM analysis also yielded interesting results. Compared with the control without CCEO, CCEO decreased the number of *E. coli* cells and disrupted biofilm formation (Figures 5A–C). After treatment with 4 μL/mL CCEO, the morphology of *E. coli* cells was destroyed and it was difficult to find intact *E. coli* cells in the microscopic field after exposure for 6 h (Figure 5C).

### Effect of CCEO on the Viability of *E. coli* During Biofilm Formation

**FIGURE 6:**
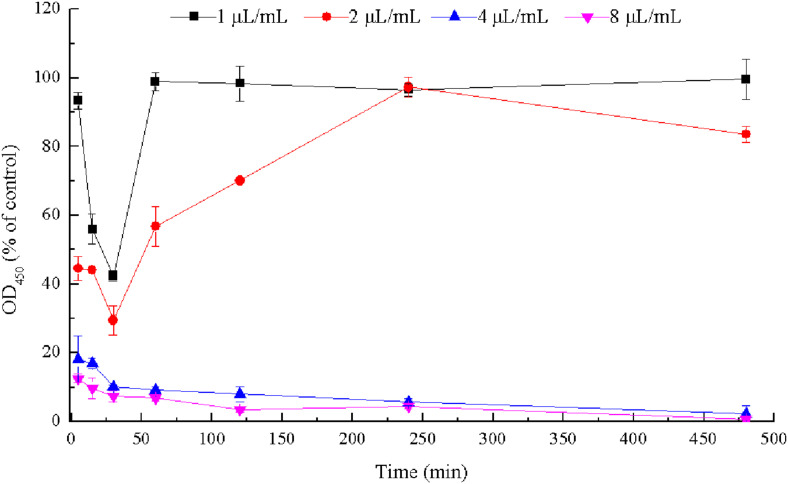
Time-kill curves of *C. camphora* essential oil (CCEO) at 1, 2, 4, or 8 μL/mL against *E. coli* ATCC 25922 biofilms. Data represents the mean ± SD of three independent experiments.

**FIGURE 7:**
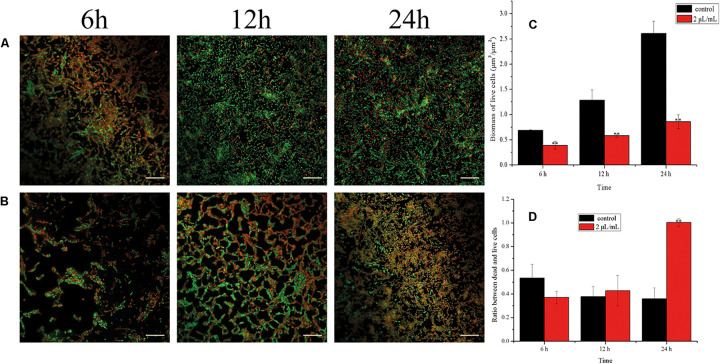
Analyses of live/dead bacteria in *E. coli* ATCC 25922 biofilms: **(A)** Confocal laser scanning microscopy (CLSM) images of C. camphora essential oil (CCEO) at 0 μL/mL for 6, 12 and 24 h; **(B)** CLSM images of CCEO at 2 μL/mL for 6, 12 and 24 h; Green, live bacteria, red, dead bacteria; Images from three independent replicates with 20 μm bars are representative; **(C)** Biomass of live cells in bilfilm; **(D)** Ratio between dead and live cells in biofilm. A sample of 0 μL/mL CCEO was used as a control. Data represents the mean ± SD of three independent experiments, ** indicates *p* < 0.01 compared with the control at the same time.

Viable cells in biofilms of *E. coli* ATCC 25922 were detected by CCK-8 assays (Figure 6) and CLSM analysis (Figure 7). After 30 min, the viability of the bacteria in biofilms exposed to 1 μL/mL CCEO was 42.33% of the control value and that after exposure to 2 μL/mL CCEO was 29.45% of the control value. However, as the exposure time increased, the inhibitory effect of CCEO gradually weakened. When 4 μL/mL CCEO was added, 82% of the biofilm formation was inhibited after exposure for 5 min, and that proportion reached 90% after exposure for 30 min. Increasing the CCEO concentration to 8 μL/mL had a similar effect as the exposure to 4 μL/mL CCEO on the kinetics of biofilm killing. These effects appeared to be dosage dependent up to 4 μL/mL CCEO. Fast killing occurred during the first 30 min, and then, the rate of killing decreased in a time-dependent manner for 4 and 8 μL/mL CCEO (Figure 6). In the CLSM images, the morphology of the *E. coli* biofilms treated with 2 μL/mL CCEO was disrupted compared with that of the untreated group (control) (Figures 7A,B). The biomass of live cells was significantly reduced after CCEO treatment compared with the control at the same time (*p* < 0.01) (Figure 7C). The ratio of dead/live cells was significantly increased after treatment with CCEO at 24 h (*p* < 0.01) (Figure 7D).

## Discussion

*Cinnamomum camphora* essential oil has been shown to exhibit medicinal activities, such as antimicrobial, insecticidal, anti-inflammatory and antioxidant activities. The obtained MICs and MBCs confirmed the high susceptibility of clinical strains to CCEO. *E. coli* biofilm formation can cause antimicrobial resistance and is closely related to persistent *E. coli* infection (Venkatesan et al., 2015; Ferris et al., 2016). In recent years, studies have shown that natural products affect *E. coli* biofilm formation (Jagani et al., 2009; Serra et al., 2016; Kang et al., 2017). In this context, we showed that CCEO efficiently kills *E. coli* in both suspension and biofilms. The MIC of CCEO obtained in this study was similar to the MIC of CCEO against *E. coli* found by Zhou et al. (2016). However, this value was far lower than the concentration required for antifungal activity against *Colletotrichum gloeosporioides*, *Botrytis cinerea*, and *Fusarium graminearum* reported by Wang et al. (2017). This discrepancy might be partly explained by the fact that the chemical compositions of different CCEOs are not the same, and different CCEOs have different inhibitory effects on bacteria, with a stronger inhibitory effect on gram-negative bacteria than on gram-positive bacteria (Jantan and Goh, 1992; Pelissier et al., 1995; Shi et al., 2013; Zhou et al., 2016). Our data showed that the MBICs were similar to the MBCs and that the MBECs were almost twice as high as the MICs (details in Supplementary Table S1). Most antibiotics are up to 1000 times less efficient against bacteria in biofilms than against those in suspension (Melchior et al., 2006), which makes CCEO a very promising antibacterial agent. For the potential clinical use of CCEO, it is important to establish the kinetics of its action against bacteria in suspension and in biofilms.

Time-kill curves and FCM analysis were used to confirm the antibacterial mode of CCEO. In FCM analysis, the fluorescent stains SYTO 9 and PI were used to evaluate microbial viability. SYTO 9 can combine with nucleic acids in all bacterial cells to emit green fluorescence, while PI penetrates only damaged bacterial membranes and combines with DNA from dead bacteria to emit red fluorescence. When SYTO 9 and PI are both present, SYTO 9 fluorescence is reduced (Oliveira et al., 2015). Our results indicated that CCEO can effectively kill *E. coli* in a dose-dependent manner, with the fastest killing occurring during the first 5 min. The antibacterial mode of action is consistent with that of tea tree oil (Kwieciński et al., 2009). The lowest level in viable bacteria was observed at approximately 1 h after treatment with subinhibitory concentrations of CCEO in our study. This finding is consistent with an early study on the vapor-phase antibacterial action of essential oil from *C. camphora* var. linaloofera Fujita (Wu et al., 2019). These findings may be a result of the bacteria being in the lag phase in the first 1 h; the damage of most bacteria caused by subinhibitory concentrations of CCEO could be gradually recovered. After entering the logarithmic phase, bacteria grow rapidly with steady geometric progression. When the rate of bacterial growth exceeded the rate of CCEO-mediated killing, the number of viable bacteria increased. After exposure to 4 or 8 μL/mL CCEO, the number of bacterial cells sharply decreased, and mainly dead cells were detected by FCM. After treatment with 4 or 8 μL/mL CCEO for 4 h, the number of bacterial cells decreased to the lowest level, with mostly viable cells detected, and the number of bacterial cells increased after 24 h based on our FCM results. This finding indicated that the killing cells were disrupted at 4 h after treatment with CCEO, and the effectiveness of CCEO decreased over time. Therefore, we speculated that the pharmacodynamic time of CCEO was less than 24 h. After treatment with 4 or 8 μL/mL CCEO, viable bacterial cells were detected by FCM, but no cells were detected by the CFU assay. One reason may be that the number of viable bacteria was very low, approximately 1 CFU/mL or even lower, at this time point, while the detection limit of the CFU assay was 10 CFU/mL. In addition, a few *E. coli* cells might exhibit sublethal injury at this time point and may then begin to multiply after recovery when the effect of CCEO is reduced (Lewis, 2007; Spilimbergo et al., 2010). Treatment with plant essential oils, such as carvacrol and citral, for 4–6 h resulted in the maximum proportion of bacterial sublethal injury (Somolinos et al., 2008; Silva et al., 2015). Thus, it is necessary to administer CCEO twice in a 24 h period in clinical use.

*Escherichia coli* biofilms show five stages of development: initial adhesion/attachment to the substrate, irreversible attachment, early development, biofilm maturation, and biofilm dispersion. The early stages of biofilm formation play an important role in the establishment of biofilms on a contact surface because these stages represent the commitment of free-living planktonic bacterial cells to a coordinated biofilm mode of survival (Karunakaran and Biggs, 2011). CCEO degraded the cell membrane and leaded to the leakage of cytoplasmic materials. Bacterial killing occurred during the first 1 h after exposure to CCEO, and we speculated that the inhibitory effect of CCEO on biofilms also appeared in the early stage. Thus, we tested whether CCEO can inhibit *E. coli* biofilm formation in the present study. Crystal violet assays are commonly used to detect the formation of bacterial biofilms. In this study, the crystal violet assay results showed that CCEO significantly decreased biofilm formation by *E. coli* in a dose-dependent manner. Our SEM results were consistent with this finding, indicating that CCEO strongly inhibited *E. coli* biofilm formation. We also found that 4 μL/mL CCEO could disrupt the biofilm and inhibit *E. coli* cell growth. A similar finding was reported by Kwieciński et al. (2009), who found that tea tree oil (1%) disrupted the biofilm of *S. aureus* due not only to bacterial killing but also partly to extracellular matrix damage and subsequent removal of the biofilm from the surface. Cui et al. (2016) observed that clove oil effectively inhibited the biofilm of *E. coli* O157:H7; clove oil causes physiological and morphological changes in cells and leads to the loss of intracellular constituents and the death of bacterial cells. Wu et al. (2019) reported vapor-phase antibacterial action of the essential oil from *C. camphora* var. linaloofera Fujita against *E. coli*; this oil partly degraded the cell membrane and increased membrane permeability, resulting in leakage of cytoplasmic materials and prominent distortion and shrinkage of the bacterial cells. Chen et al. (2020) reported that the essential oil from the leaves of *C. camphora* (Linn.) Presl inhibited Methicillin-resistant *S. aureus* via damaging cell membranes and disturbing the amino metabolism. We also found the cell membranes of *E. coli* were partially ruptured and the cytoplasmic materials leaked after the treatment with 2 μL/mL CCEO (details in Supplementary Figure S1). Thus, we speculated that CCEO could penetrate the *E. coli* biofilm and inhibit bacterial proliferation to reduce the biofilm formation.

The biofilm yield depends on the number of bacteria residing in the biofilm. To quantify the viable count, researchers commonly use mechanical methods (e.g., ultrasonication or scraping) to separate biofilms from the solid surface to which they are adhered (Webber et al., 2015). However, these methods may damage *E. coli* cells. In this study, we performed quantitative analysis of live bacterial cells in biofilms by CCK-8 staining. This method determines the viability of bacterial biofilms based on the reduction of tetrazolium salts to formazan by viable metabolizing bacteria (Berk et al., 2012; Chen et al., 2019). Using this method, our study showed that 4 μL/mL CCEO could inhibit the metabolism of *E. coli* in biofilms, and the rate of killing was concentration dependent up to 4 μL/mL CCEO. Further increases in the CCEO concentration did not significantly accelerate bacterial killing in biofilms. A high respiratory rate of the biofilms was observed after treatment with 2 μL/mL CCEO, particularly at 4 h. This finding is in contrast to the data of crystal violet assay suggesting that 2 μL/mL CCEO significantly inhibited the biofilm. This might be explained by the different methods of viability testing and biofilm formation and the different objects to study. The crystal violet assay was used to detect the effect of CCEO on the biofilm formation of *E. coli*. Not only bacteria but also extracellular polymeric substances such as exopolysaccharides in biofilms were detected by the crystal violet assay. The CCK-8 method determines the respiratory rate of bacteria in the biofilms, including many already lethally injured cells that might still exhibit metabolism. Bacterial viability was inhibited before exposure to CCEO for 4 h, and it was difficult for the injured bacterial cells to secrete the same amount of extracellular polymeric substances like the untreated control. Thus, the inhibition effect of CCEO measured by the crystal violet assay was not consistent with the results of CCK-8. The number of live bacteria in the biofilm was lowest at 30 min, which was inconsistent with the findings at 1 h in suspension. This discrepancy is possibly due to the different growth curves of bacteria in suspension and in biofilms. Furthermore, CLSM and SYTO 9 and PI staining are commonly used to visualize cell viability in biofilms (Kulshrestha et al., 2016). CLSM images also indicated that the number of viable biofilm cells was significantly reduced and the ratio of dead/live cells was significantly increased following treatment with 2 μL/mL CCEO. These results suggested that CCEO killed *E. coli* and inhibited bacterial proliferation to reduce the biofilm formation.

Overall, CCEO exhibited significant antibacterial activity against *E. coli* in suspension and biofilms, two states frequently encountered in living organisms. The MBECs of CCEO against clinical *E. coli* strains were generally two times higher than the MICs. CCEO killed *E. coli* quickly and effectively at 4 μL/mL and caused the destruction of *E. coli* biofilms. The effect of CCEO on *E. coli in vivo* and the mechanism of action of CCEO against *E. coli* biofilms need to be further studied.

## Data Availability Statement

The original contributions presented in the study are included in the article/supplementary material, further inquiries can be directed to the corresponding author.

## Ethics Statement

The animal study was reviewed and approved by Animal Ethics Committee of Lanzhou Institute of Husbandry and Pharmaceutical Sciences of CAAS.

## Author Contributions

LW, KnZ, KiZ, JF, JZ, and JEL designed and performed the lab experiments and analyzed the data. LW, GW, and ZQ designed the figures and analyzed the image data. XW and JXL discussed the results and supervised the project. LW wrote the manuscript in consultation with KnZ, KiZ, JZ, GW, ZQ, XW, and JXL. All authors contributed to the article and approved the submitted version.

## Conflict of Interest

The authors declare that the research was conducted in the absence of any commercial or financial relationships that could be construed as a potential conflict of interest.

## Footnotes

1 www.comstat.dk
