# Supplementary material for: Antibacterial Activity of Cinnamomum camphora Essential Oil on Escherichia coli During Planktonic Growth and Biofilm Formation
Source: Front Microbiol. 2020 Nov 12;11:561002. doi: 10.3389/fmicb.2020.561002 (PMC7693543; doi:10.3389/fmicb.2020.561002)
Supplement: Supplementary file 1 [file Data_Sheet_1.docx]

Supplementary Material

**Antibacterial activity of *Cinnamomum camphora* essential oil on *Escherichia coli* during planktonic growth and biofilm formation**

Lei Wang, Kang Zhang, Kai Zhang, Jingyan Zhang, Jingjing Fu, Jie Li, Guibo Wang, Zhengying Qiu, Xuezhi Wang, Jianxi Li ^*^

Key Lab of Veterinary Pharmaceutical Development, Ministry of Agriculture and Rural Affairs; Engineering and Technology Research Center of Traditional Chinese Veterinary Medicine, Gansu Province; Lanzhou Institute of Husbandry and Pharmaceutical Sciences of Chinese Academy of Agricultural Sciences, Lanzhou, China

*** Correspondence:** Jianxi Li, phD, Professor

Lanzhou Institute of Husbandry and Pharmaceutical Sciences of Chinese Academy of Agricultural Sciences. 335 Jiangouyan, Xiaoxihu, Lanzhou, 730050, Gansu, China.

**E-mail:** [lzjianxil@163.com](mailto:lzjianxil@163.com)

**Supplementary Table S1.** MIC, MBC, MBIC and MBEC of *C. camphora* essential oil (CCEO) against isolates of *E. coli* isolated from dairy cows with clinical endometritis

| **Columns of isolates** | **Accession numbers** | **Minimum inhibitory concentration (MIC, μL/mL)** | **Minimum bactericidal concentration (MBC, μL/mL)** | **Minimum biofilm inhibitory concentration (MBIC, μL/mL)** | **Minimum biofilm eradication concentration (MBEC, μL/mL)** |
| --- | --- | --- | --- | --- | --- |
| DT003 | MW025989 | 2.00±0.00 | 2.00±0.00 | 2.67±1.15 | 3.33±1.15 |
| DT005 | MW025990 | 2.67±1.15 | 3.33±1.15 | 3.33±1.15 | 4.00±0.00 |
| DT012 | MW025991 | 2.67±1.15 | 3.33±1.15 | 3.33±1.15 | 3.33±1.15 |
| DT122 | MW025992 | 2.00±0.00 | 4.00±0.00 | 3.33±1.15 | 4.00±0.00 |
| DT211 | MW025993 | 2.00±0.00 | 2.00±0.00 | 2.67±1.15 | 3.33±1.15 |
| DT231 | MW025994 | 2.00±0.00 | 2.00±0.00 | 3.33±1.15 | 3.33±1.15 |
| DT251 | MW025995 | 2.00±0.00 | 2.00±0.00 | 2.00±0.00 | 3.33±1.15 |
| DT271 | MW025996 | 2.00±0.00 | 4.00±0.00 | 3.33±1.15 | 4.00±0.00 |
| QH029 | MW025997 | 4.00±0.00 | 4.00±0.00 | 4.00±0.00 | 4.00±0.00 |
| QH034 | MW025998 | 8.00±0.00 | 8.00±0.00 | 5.33±2.31 | 10.67±4.62 |
| QH142 | MW025999 | 8.00±0.00 | 13.33±4.62 | 5.33±2.31 | 13.33±4.62 |
| QH243 | MW026000 | 4.00±0.00 | 8.00±0.00 | 5.33±2.31 | 8.00±0.00 |
| QH253 | MW026001 | 4.00±0.00 | 4.00±0.00 | 4.00±0.00 | 5.33±2.31 |
| QH273 | MW026002 | 5.33±2.31 | 6.67±2.31 | 5.33±2.31 | 8.00±0.00 |
| QH274 | MW026003 | 4.00±0.00 | 4.00±0.00 | 4.00±0.00 | 5.33±2.31 |
| QH334 | MW026004 | 4.00±0.00 | 8.00±0.00 | 4.00±0.00 | 8.00±0.00 |
| QH362 | MW026005 | 4.00±0.00 | 5.33±2.31 | 4.00±0.00 | 5.33±2.31 |
| QWC0999 | KJ577272 | 6.67±2.31 | 6.67±2.31 | 5.33±2.31 | 6.67±2.31 |
| QWC10083 | KJ577269 | 4.00±0.00 | 8.00±0.00 | 5.33±2.31 | 8.00±0.00 |
| QWC10099 | KJ577271 | 8.00±0.00 | 13.33±4.62 | 10.67±4.62 | 16.00±0.00 |
| QWC1041 | KJ577270 | 6.67±2.31 | 8.00±0.00 | 5.33±2.31 | 8.00±0.00 |
| QWC11095 | KJ577268 | 5.33±2.31 | 8.00±0.00 | 5.33±2.31 | 8.00±0.00 |
| SA001 | MW026007 | 4.00±0.00 | 6.67±2.31 | 5.33±2.31 | 8.00±0.00 |
| SA007 | MW026008 | 4.00±0.00 | 5.33±2.31 | 5.33±2.31 | 5.33±2.31 |
| SA021 | MW026009 | 3.33±1.15 | 4.00±0.00 | 4.00±0.00 | 4.00±0.00 |
| SA066 | MW026010 | 3.33±1.15 | 4.00±0.00 | 4.00±0.00 | 4.00±0.00 |
| SA080 | MW026011 | 4.00±0.00 | 4.00±0.00 | 5.33±2.31 | 5.33±2.31 |
| SA092 | MW026012 | 4.00±0.00 | 4.00±0.00 | 5.33±2.31 | 5.33±2.31 |
| SA120 | MW026013 | 5.33±2.31 | 5.33±2.31 | 5.33±2.31 | 6.67±2.31 |
| SA123 | MW026014 | 3.33±1.15 | 3.33±1.15 | 4.00±0.00 | 4.00±0.00 |
| SA202 | MW026015 | 4.00±0.00 | 4.00±0.00 | 4.00±0.00 | 5.33±2.31 |
| SA411 | MW026016 | 4.00±0.00 | 4.00±0.00 | 4.00±0.00 | 4.00±0.00 |
| SB004 | MW026018 | 4.00±0.00 | 4.00±0.00 | 4.00±0.00 | 6.67±2.31 |
| SB032 | MW026019 | 4.00±0.00 | 4.00±0.00 | 4.00±0.00 | 4.00±0.00 |
| SB078 | MW026020 | 8.00±0.00 | 8.00±0.00 | 5.33±2.31 | 8.00±0.00 |
| SB222 | MW026021 | 4.00±0.00 | 6.67±2.31 | 4.00±0.00 | 8.00±0.00 |
| SB223 | MW026022 | 4.00±0.00 | 4.00±0.00 | 5.33±2.31 | 5.33±2.31 |
| SB311 | MW026023 | 5.33±2.31 | 5.33±2.31 | 5.33±2.31 | 6.67±2.31 |
| SB041 | MW026024 | 4.00±0.00 | 4.00±0.00 | 4.00±0.00 | 5.33±2.31 |
| QH404 | MW026006 | 4.00±0.00 | 4.00±0.00 | — | — |
| SA006 | MW026017 | 4.00±0.00 | 4.00±0.00 | — | — |
| SB110 | MW026025 | 4.00±0.00 | 5.33±2.31 | — | — |
| SB211 | MW026026 | 6.67±2.31 | 6.67±2.31 | — | — |
| SB922 | MW026027 | 5.33±2.31 | 6.67±2.31 | — | — |

“—” indicates undetectable.

**Supplementary Figures**

**1 Materials and Methods**

**1.1 Transmission Electron Microscopy (TEM) Analysis**

The effects of CCEO on the morphological changes occurring in *E. coli* cells were determined by TEM. *E. coli* ATCC 25922 suspensions (1 × 10^6^ CFU/mL) were treated with CCEO at 0 μL/mL (control) and 2 μL/mL for 24 h at 37°C. After treatment, the cells of two groups were separately centrifuged at 5000 rpm for 10 min, the medium was removed, and the cells were resuspended in 0.5% (v/v) glutaraldehyde for 10 min. Then these cells were centrifuged at 10000 rpm for 10 min. These cells were fixed with 3.0% (v/v) glutaraldehyde overnight at 4°C and dehydrated with serial acetone (30%, 50%, 70%, 80%, 90%, 95%, 100%, 100%, and 100%). Subsequently, the samples were permeated, embedded and sliced. Finally, they were successively dyed with uranium acetate and lead citrate and imaged with TEM (H-600IV, Hitachi, Japan).

**2 Results**

**2.1 Effect of CCEO on *E. coli* observed by TEM**

The changes of internal structure of *E. coli* ATCC25922 after treatment with CCEO were examined by TEM. The cell walls in the control were closely attached to the cell membranes and the cytoplasm was evenly distributed (Fig. S1A). After the treatment with 2 μL/mL CCEO, the cell membranes were partially ruptured and the cytoplasmic materials leaked. The cell membranes were separated from the cell walls, the cytoplasm became unevenly distributed and even some cavities appeared inside the cells (Fig. S1B).


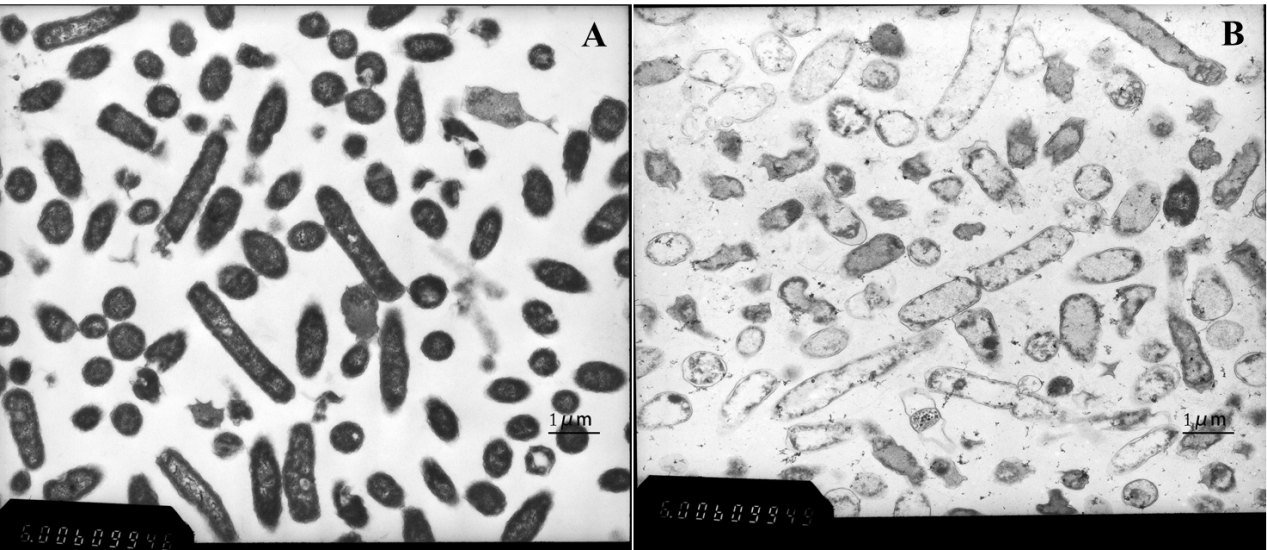


**Supplementary Figure S1.** Transmission Electron Microscopy (TEM) images of *E. coli* ATCC 25922: (A) control; (B) treated with *C. camphora* essential oil (CCEO) at 2 µL/mL for 24 h. A sample of 0 μL/mL CCEO was used as a control.
